# Emission-wavelength-dependent photoluminescence decay lifetime of N-functionalized graphene quantum dot downconverters: Impact on conversion efficiency of Cu(In, Ga)Se_2_ solar cells

**DOI:** 10.1038/s41598-019-47068-w

**Published:** 2019-07-25

**Authors:** Firoz Khan, Jae Hyun Kim

**Affiliations:** 1 Center of Research Excellence in Renewable Energy (CoRERE), King Fahd University of Petroleum and Minerals (KFUPM), Dhahran, 31261 Saudi Arabia; 2 Smart Textile Convergence Research Group, Daegu Gyeongbuk Institute of Science & Technology (DGIST), 333 Techno Jungang-Daero, Hyeonpung-Myeon, Dalseong-Gun, Daegu, 42988 Republic of Korea

**Keywords:** Environmental chemistry, Energy

## Abstract

Graphene quantum dots (GQDs) have several advantages over inorganic quantum dots owing to their beneficial properties. Recently, GQDs have been used as downconverters in photovoltaic devices. However, the application of GQDs in most emergent thin-film-based Cu(In, Ga)Se_2_ (CIGS) photovoltaic cells is limited because of either low photoluminescence (PL) quantum yield (QY) or a small Stokes shift (Δλ). Therefore, GQDs with an ultrahigh QY and large Δλ are essential to realizing the two emergent fields, i.e., the application of GQDs in CIGS photovoltaic solar cells. In this regard, we synthesized nitrogen-functionalized GQDs (NGQDs) with an ultrahigh QY (77–99%) and a large Δλ (95–155 nm) via tailoring of the nitrogen and oxygen moieties. The NGQDs were applied in CIGS solar cells to evaluate their downconversion efficiency. Our study shows that the emission wavelength (λ_em_)-dependent photoluminescence decay lifetime (τ_em_) determines the down-conversion efficiency of the nitrogen-functionalized graphene quantum dots. With the increase in *τ*_em_ at λ_em_ > 500 nm, the conversion efficiencies of the NGQDs coated-CIGS solar cells increased by 12.22%. Thus, the increase in *τ*_em_ at λ_em_ > 500 nm significantly increased the maximum current output and thus enhanced the solar-cell performance.

## Introduction

Recently, carbon-based quantum dots (QDs) and graphene QDs (GQDs) have received considerable attention due to their advantageous characteristics^1^. GQDs are attractive owing to the unique broadband-tunable photoluminescent (PL) properties, their excellent biocompatibility, high photostability, low toxicity, low cost, and due to the abundance of the raw materials used to create them^2,3^. They have promising applications in bioimaging^2,4^, biosensing^5,6^, environmental monitoring^7^, light-emitting diodes^8^, and photovoltaic (PV) devices^9,10^.

Previously, several research groups used GQDs as photon downconverters in silicon-based PV devices^9,10^. A downconverter absorbs photons of at short wavelengths (*λ*) and emits photons at longer *λ* levels. The downconversion efficiency (*η*_*dc*_) of a downconverter can be determined by Eq. (1),

1 $${\eta }_{dc}\,=\,{N}_{phe}/{N}_{pha}$$

where N_phe_ denotes the number of useful emitted photons which can be absorbed by PV materials to generate current, and N_pha_ is the total number of absorbed photons by downconverters.

The value of η_dc_ is essentially governed by the PL quantum yield (QY) and the Stokes shift (*Δλ*). For application in PV devices, GQDs should exhibit a high PL QY in conjunction with a large *Δλ* value. Unfortunately, most of the reported GQDs are not suitable for application in prominent thin-film-based Cu(In, Ga)Se_2_ (CIGS) PV cells^1,3,9–12^.

On the other hand, commercial interest is beginning to shift toward thin-film cells owing to their low material and manufacturing costs^13^. However, there is still a large difference between the experimentally achieved and theoretical efficiency (∼33%, *E*_g_ ≈ 1.15 eV)^14^. The parasitic absorption losses in the ZnO and CdS layers weaken the short-*λ* response, representing the predominant hurdles in the quest to realize high-performance CIGS solar cells^14–17^. The poor short-*λ* response is inherently linked to the interaction of short-*λ* photons with the front layers of solar cells. A luminescent downconverter (LDC) can be used to improve the short-*λ* response. For down-conversion, several LDC materials have been investigated, including GQDs^1,3,9–11^, inorganic QDs^18–21^, organic dyes^22,23^, and rare-earth ions/complexes^24–26^. However, these are not convenient downconverters owing to one or more limitations, e.g., a low PL QY, a narrow absorption band, a small value of *Δλ*, low absorption coefficients, low stability, and toxicity. In addition, these materials are generally expensive^27^. Therefore, we previously investigated a novel approach by which to produce N-functionalized GQDs (NGQDs) rapidly. The produced NGQDs exhibited an ultrahigh PL QY and a large *Δλ*, which are mandatory for enhancing the performance capabilities of CIGS solar cells. Furthermore, the produced NGQDs was used to coat a layer onto the top of a CIGS solar cell, and the ensuing performance enhancement was studied^28^.

In the present study, we tuned the synthesis parameters of NGQDs to achieve an ultrahigh PL QY along with a very large value of *Δλ* (PL emission peak at *λ*_*em*_ > 500 nm) via tailoring the fractions and concentrations of the N and O moieties. A new parameter — the emission-wavelength-dependent PL decay lifetime (*τ*_*em*_) — was introduced, which greatly affects the down-conversion efficiency of NGQDs in CIGS solar cells. NGQDs with various *τ*_*em*_ values were applied to CIGS solar cells to evaluate their downconversion performance. The PL decay spectra at various emission wavelengths were recorded and used to determine the value of τ_em_. To the best of our knowledge, the relationship between the emission-wavelength-dependent PL decay lifetime and the CIGS solar-cell performance was established for the first time [10].

## Results

Various NGQDs were synthesized by controlling the polyethyleneimine (PEI)/graphene oxide (GO) weight ratio, synthesis temperature, and synthesis time. The synthesis parameters of the NGQD samples and the PL QYs are presented in Table S1. Only four samples (#3, #9, #11, and #12) exhibited a PL QY of >75%; therefore, we used these samples for a more detailed study. The other NGQDs for which PL QY < 75% do not have much of an impact on enhancing the performance of CIGS solar cells. Therefore, a detailed study of NQGDs for which the PL QY < 75% was not done.

**Figure 1:**
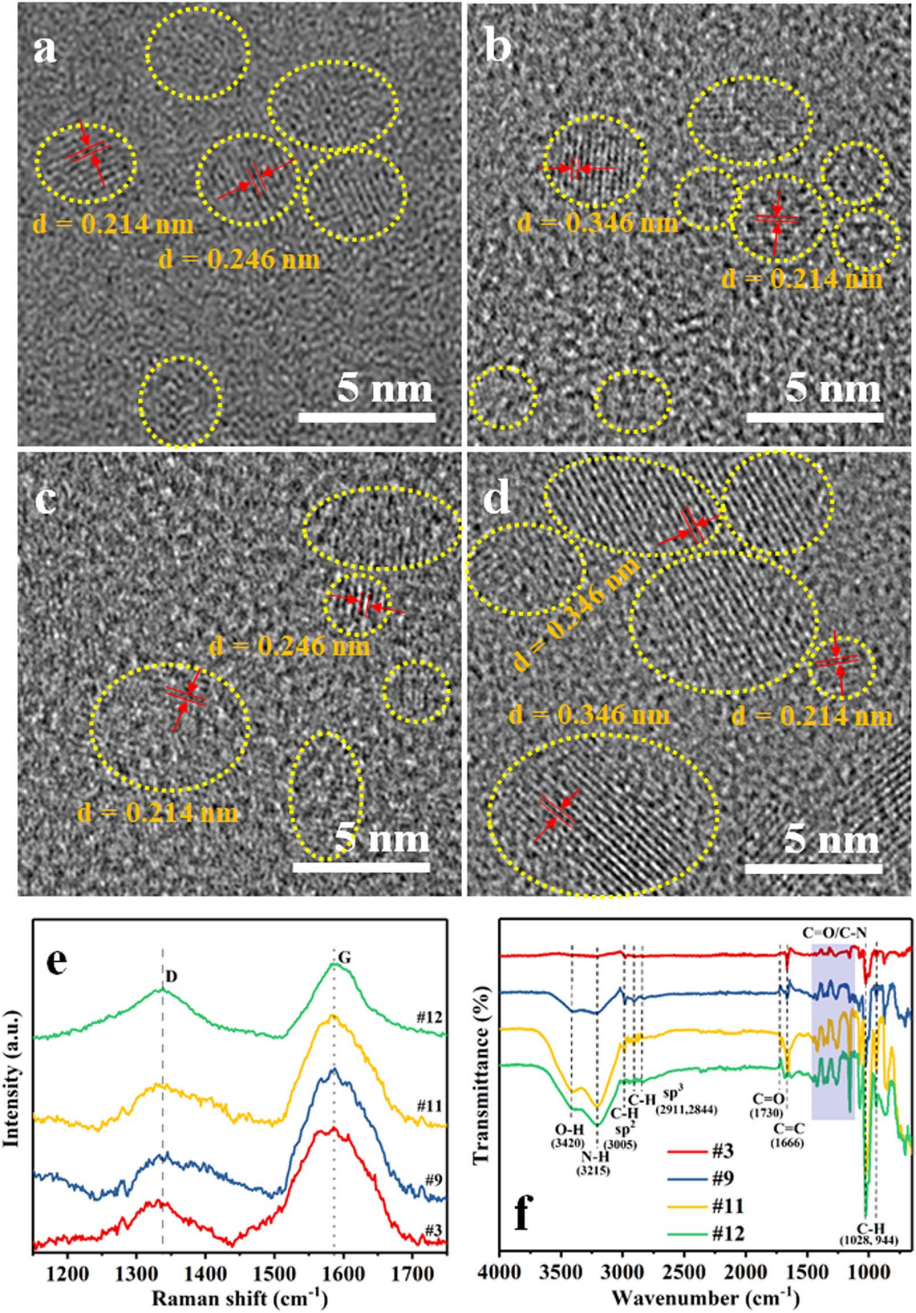
HRTEM images of NGQD samples (**a**) #3, (**b**) #9, (**c**) #11, and (**d**) #12. (**e**) Raman spectra and (**f**) FTIR transmittance spectra of the samples.

High-resolution transmission electron microscopy (HRTEM) images of the NGQDs (#3, #9, #11, and #12) are shown in Fig. 1(a–d). These images show lattice fringes for all samples, indicating high crystallinity of the synthesized NGQDs. The spacings between the fringes were determined to be 0.346 and 0.214 nm, corresponding to the (002) and (100) planes of graphite. However, the calculated d-spacings between the basal planes of the NGQDs are slightly wider than those of graphite (d_002_ = 0.334 nm and d_100_ = 0.213 nm) owing to the presence of functional groups^12^. The spacing of 0.246 nm corresponds to the lattice constant of graphite (a = 0.246 nm). Figure S1 shows the size distribution of the NGQD samples. The maximum fractions of the NGQD size obtained for samples #3, #9, #11 and #12 are 3.0 nm, 2.1 nm, 1.8 nm, and 3.9 nm, respectively. The Raman spectra of the NGQDs are shown in Fig. 1(e). The peaks at 1,346 and 1,587 cm^−1^ are assigned to the characteristic D and G bands, respectively^10,11^. Thus, the spectroscopic analysis confirms the formation of NGQDs. The Fourier transform infrared (FTIR) spectra of the NGQD samples are shown in Fig. 1(f). The peaks at 3,420 and 3,215 cm^–1^ are attributed to the stretching vibrations of O–H and N–H, respectively^3^. The bond signatures of sp^2^ C–H (C=C–H) at 3,005 cm^–1^, sp^3^ C–H (C–C–H) at 2,911 and 2,844 cm^–1^, and C=O at 1,730 cm^–1^ are observed for samples #9, #11 and #12^1,3,29^. The absorption peaks due to the bond signatures of C=C at 1,666 cm^–1^ and C–H at 1,028 and 944 cm^–1^ are also observed for all of the samples^30^. The peaks at 1,028 cm^–1^ (C–H in-plane bending vibration) and 944 cm^–1^ (H out-of-plane wagging) are attributed to aromatic moieties^30^. The several peaks found in the region of 1,500–1,100 cm^–1^ are assigned to the C=C and C–N stretching modes^31,32^.

**Figure 2:**
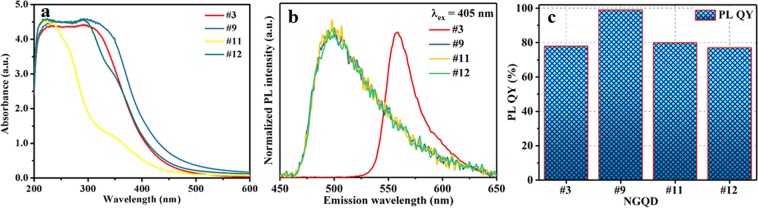
(**a**) UV absorbance spectra, (**b**) PL spectra emission spectra at λ_ex_ = 405 nm, and (**c**) PL QY of NGQD samples #3, #9, #11, and #12.

The ultraviolet-visible (UV-Vis) absorption spectra of the NGQD solutions were measured in the λ range of 200–600 nm. Broad absorption was obtained all four samples above (Fig. 2(a)). The strong absorption at *λ* < 500 nm is attributed to the encompassment of several characteristic absorption bands. Typically, the *π* → π* transitions of C=C bonds cause absorption in the deep UV region^33,34^. However, the absorption in the low *λ* range (270–420 nm) corresponds to the C*π* → Nπ*/C*π* → Oπ* transitions of C–N/C = N and C=O^35^. N doping introduces a new energy level (Nπ*), which is responsible for the aforementioned new peaks. In addition, the absorption peaks due to the C*π* → Oπ* transition shift toward higher wavelengths with N doping^36^. Thus, the NGQDs exhibit strong absorption for λ < 500 nm, indicating that the produced NGQDs are extremely applicable for CIGS-based solar cells. Moreover, the absorption spectra of NGQDs synthesized with PEI/GO weight ratios of 10%, 20%, and 50% at 200–350 °C are shown in Fig. S2(a–c). The samples prepared with ratios of 10% and 20% at 350 °C exhibit weak absorption. The absorption spectra of the samples synthesized with a ratio of 20% at 300 °C for 15, 30, and 90 min are presented in Fig. S2(d).

Figure 2(b) presents the PL emission spectra recorded with *λ*_ex_ = 405 nm. The PL emission range for NGQD samples #9, #11, and #12 is 460–650 nm (peak at ~500 nm); however, the emission range for sample #3 is 550–650 nm (peak at ~560 nm). It can be seen from Fig. 2(c) that the synthesized NGQD samples #3, #9, #11, and #12 exhibit PL QYs of 78%, 99%, 80%, and 77%, respectively. The *Δλ* values for samples #9, #11, and #12 are identical (~95 nm); however, the value for #3 is ~155 nm. The full width at half-maximum (FWHM) values for samples #3, #9, #11, and #12 are 31, 66, 66, and 66 nm, respectively. Notably, the calculated FWHM values are lower than those reported by Qu *et al*.^3^ Moreover, PL emission peaks at *λ*_*ex*_ = 379 nm were observed at emission wavelengths of 524 nm, 442 nm, 457 nm, and 458 nm for NGQD samples #3, #9, #11 and #12, respectively (Fig. S3). It can be noted that the PL peak positions of NGQDs at *λ*_*ex*_ = 379 nm are quite different at *λ*_*ex*_ = 405 nm. The similar, dependency of the PL peak position of NQGD on *λ*_*ex*_ is observed by several research groups^37–39^. It has been observed that the dependency of PL peak position of NQGD on *λ*_*ex*_ is due to the size of NGQDs (quantum confinement effect)^37^, trap states at the surface of NGQDs (functional groups/dangling bonds)^38^ and electronegativity of heteroatoms^39^. However, the proposed reasons for *λ*_*ex*_-dependent PL position is still controversial, it needs further discussion^40^.

**Figure 3:**
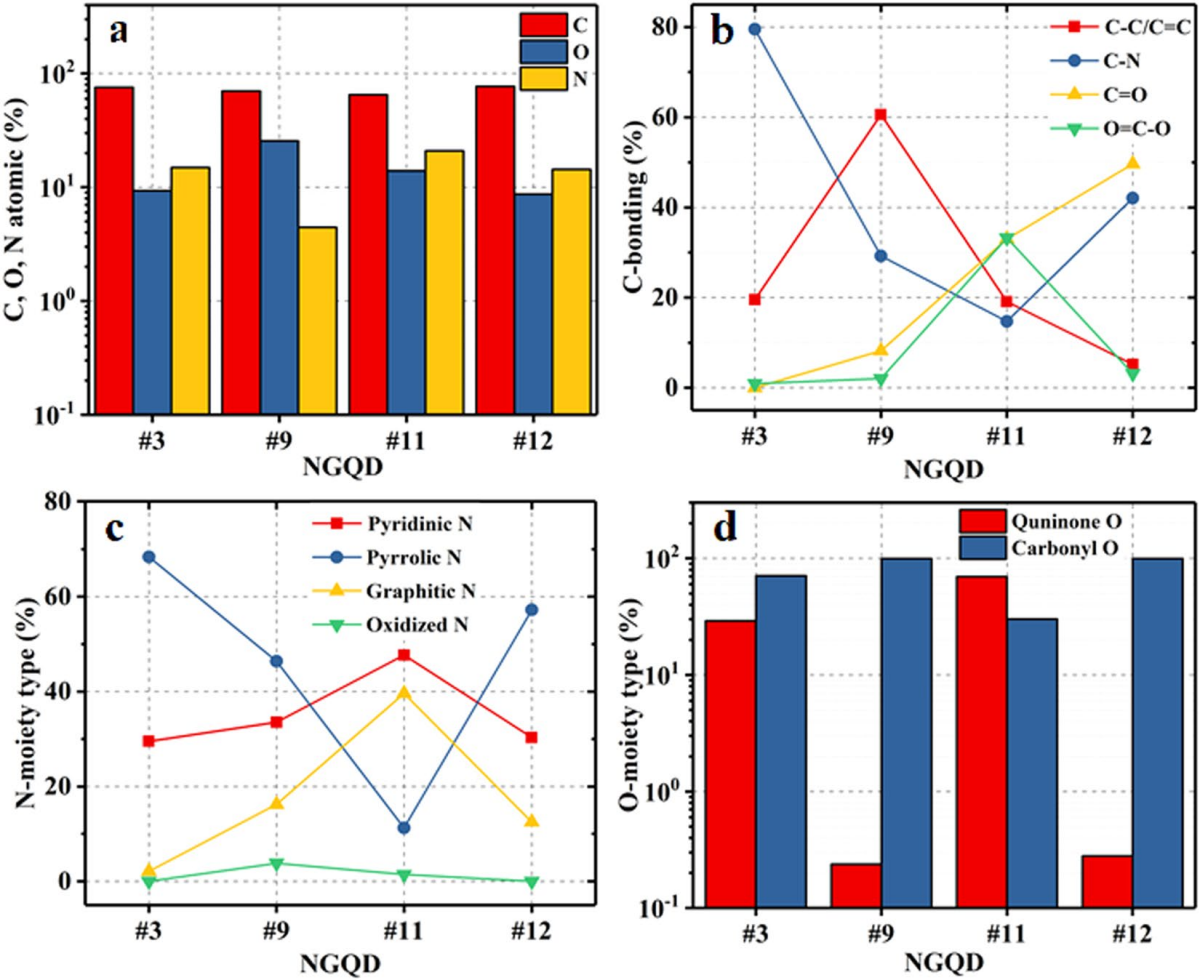
XPS analysis results: (**a**) atomic percentages of C, O, and N and proportions of (**b**) C-containing bonds, (**c**) N-moiety types, and (**d**) O-moiety types in NGQD samples #3, #9, #11, and #12.

We compared the absorption and the PL results between the NGQDs prepared in this study and those reported previously. These results are presented in Table S2. It can be seen that previously reported NGQDs have one or more flaws, specifically, low PL QYs, inappropriate absorption, and emission ranges, and low *Δλ* value. Thus, previously reported NQGDs are not applicable for use in CIGS solar cells^1,3,11^. In this study, the NGQDs are highly functionalized, and hence the PL QY, absorption, and emission bands are highly dependent on N, O moieties and C bonding (Fig. 3). With the increase in the size of NGQDs, the PL QYs was decreased from >75% to ~20%, which are not applicable for CIGS solar cells. Thus, investigation of the dependency of CIGS solar cell performance on NGQDs size is not studied.

X-ray photoelectron spectroscopy (XPS) measurements were taken to study the C-containing bonds and to determine the N moieties, O moieties, and elemental compositions. The XPS elemental survey spectra (Fig. S4) exhibit three predominant peaks attributed to C1s (~284 eV), N1s (~399 eV), and O1s (~531 eV). The atomic percentages of C, O, and N are shown in Fig. 3(a). The deconvoluted C1s spectrum exhibits four peaks conforming to C–C/C=C, C–N, C=O, and O=C–O bonds (Fig. S5)^25^, and the relative proportions of these bonds in the samples are presented in Fig. 3(b). Sample #3 exhibits the largest fraction of C–N bonds (~80%); however, the proportions of C=O and O=C–O bonds are negligible in this sample. Sample #12 shows the largest fraction of C=O bonds and sample #9 shows the largest fraction of C–C/C=C bonds (~60%). The deconvoluted N1s spectrum shows four peaks which can be assigned to pyridinic, pyrrolic, graphitic, and oxidized N (Fig. S6)^26^. The proportions of the various types of N moieties in the NGQD samples are presented in Fig. 3(c). The deconvoluted O1s spectrum exhibits two peaks corresponding to quinone and carbonyl O (Fig. S7). Figure 3(d) shows that the fraction of quinone O in samples #9 and #12 is negligible^24^.

## Discussion

**Figure 4:**
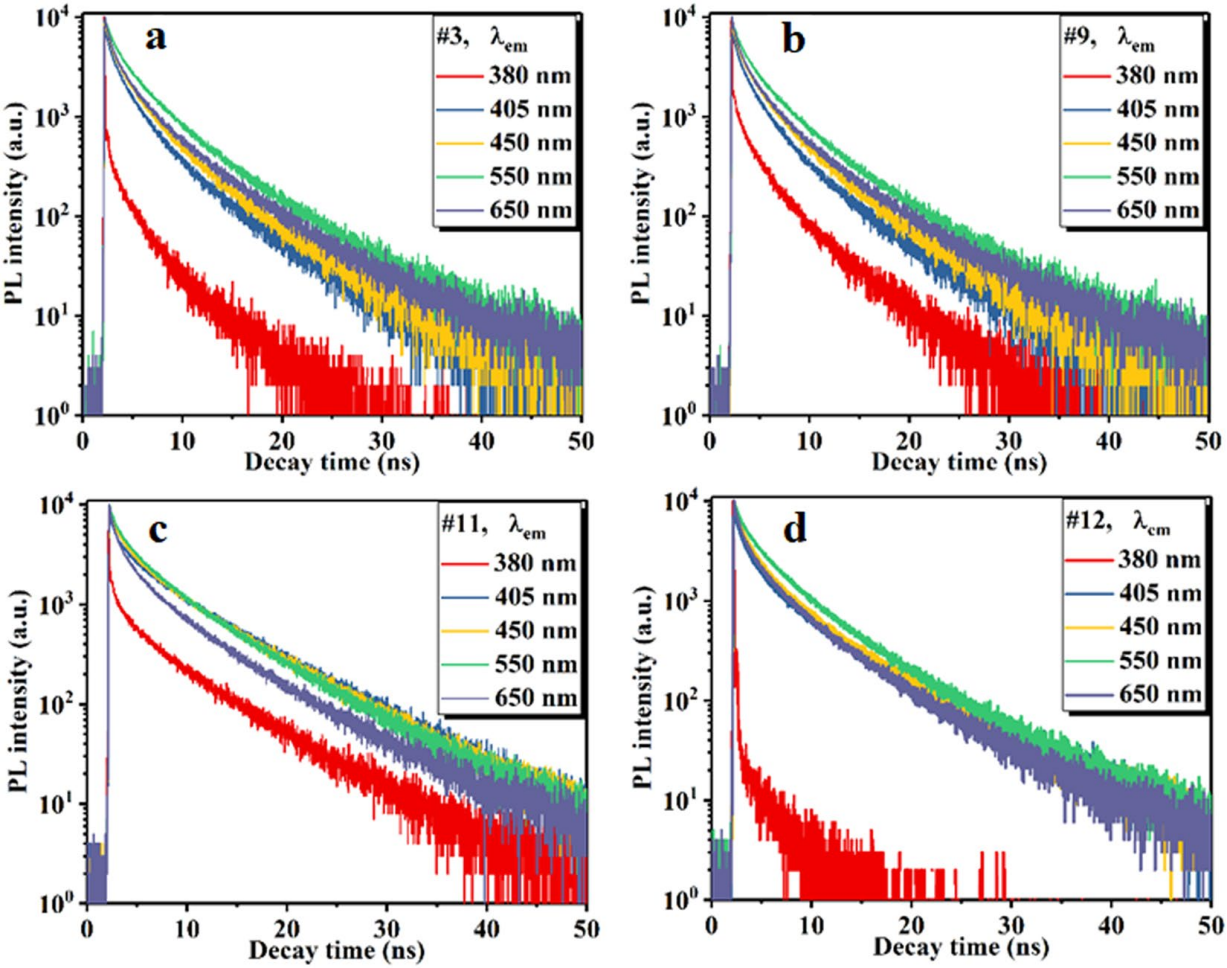
PL decay spectra at various emission wavelengths for NGQD samples (**a**) #3, (**b**) #9, (**c**) #11, and (**d**) #12.

The large fractions of C-N bonding and pyridinic N may cause the largest *Δλ* of sample #3. However, the largest fraction of C-C/C=C bonding may be responsible for the ultra-high PL QY (sample #9). The time-resolved PL decay spectra were recorded using a time-correlated single-photon counting technique at various emission wavelengths of 380, 405, 450, 550, and 650 nm for NGQDs #3, #9, #11, and #12 (Fig. 4(a–d)). The average value of *τ*_*em*_ at a given *λ*_*em*_ was determined via integration of the PL decay curve using equation (S1 of the Supplementary Information). The values of the integrated area and the calculated *τ*_*em*_ values are listed in Table S3.

**Figure 5:**
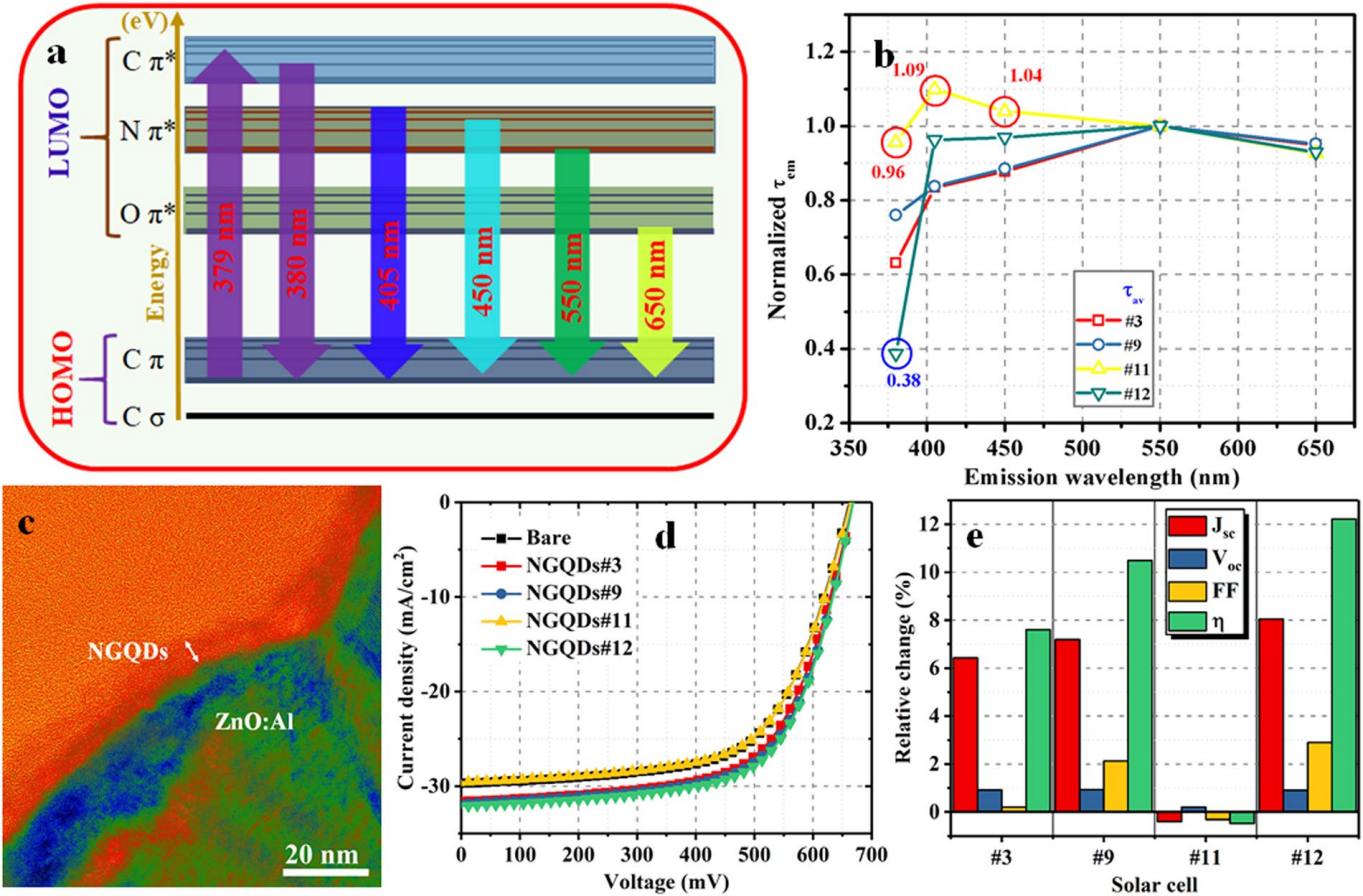
(**a**) Schematic of possible transitions at various emission wavelengths. (**b**) Normalized *τ*_em_ values of NGQD samples #3, #9, #11, and #12. (**c**) Cross-sectional HRTEM image of the ZnO:Al/NGQD interface. (**d**) *J*–*V* characteristics of NGQD-CIGS solar cells #3, #9, #11, and #12. (**e**) Relative change in the performance parameters of CIGS solar cells.

A schematic of the energy levels and the possible electron transitions responsible for the photon emission is presented in Fig. 5(a). When the NGQDs are illuminated by a light source, e.g., *λ*_ex_ = 379 nm, the photons are absorbed, resulting in the electron transitions of C*π* → Cπ*, C*π* → Nπ*, or C*π* → Oπ*. The excited electrons are deactivated via two mechanisms. One is by direct recombination after vibration relaxation due to the transition between the same energy levels, leading to PL. In this case, the PL QY can be high, but *Δλ* is low. The other mechanism is a transition through intersystem crossing (Cπ* → Nπ*, Cπ* → Oπ*), followed by vibration relaxation^36^. Finally, the recombination occurs and produces PL. Due to the large *Δλ*, the second process is very effective for downconversion (larger values of *η*_*dc*_).

The average values of *τ*_*em*_ were normalized with respect to the value at 550 nm (Fig. 5b). For samples #3, #9, and #12, the values of *τ*_*em*_ are larger at *λ*_*em*_ = 550 nm than at lower emission wavelengths (380, 405, and 450 nm). However, for sample #11, *τ*_*em*_ at the emission wavelength of 550 nm is smaller than those at 405 and 450 nm, indicating that the amount of emitted photons at *λ*_*em*_ < 550 nm is greater than that at *λ*_*em*_ = 550 nm. Thus, sample #11 cannot be used for downconversion in CIGS solar cells. The fraction of quninone O higher than that of carbonyl O may be the cause of the higher value of *τ*_*em*_ at the lower *λ*_*em*_ *(λ*_*em*_ < 550 nm) for sample #11. At *λ*_*em*_ = 380 nm, sample #12 exhibits the smallest *τ*_em_ (~38% of *τ*_*em*_ at 550 nm), while sample #11 exhibits the largest *τ*_em_ (~96% of *τ*_*em*_ at 550 nm). These findings indicate that the ratios of the number of photons emitted at *λ*_*em*_ = 380 nm to the number of photons emitted at *λ*_*em*_ = 550 nm are correspondingly 0.38 and 0.96 for samples #11 and #12. The emitted photons at a wavelength of 380 nm make no contribution to the generation of current. Therefore, the maximum loss arises for sample #11, whereas this loss is minimum for sample #12 (Fig. 5b). The value of *τ*_*em*_ for NGQDs at a particular emission wavelength indicates the effectiveness of the photon emission at that wavelength. To the best of our knowledge, this is the first report of PL-decay-lifetime-dependent photon emission efficiency and its impact on solar energy conversion. In addition to the *Δλ* and PL QY values, the decay lifetimes at slow transitions play a significant role in determining the conversion efficiency (*η*) of solar cells. At a particular wavelength, a larger τ_em_ indicates more effective photon emission. An NGQD layer with a thickness of ~4 nm was realized on the front surface of the cell (Fig. 5c). Current density–voltage (*J*–*V*) characterization was carried out under illuminated to evaluate the performance capability of the cells (Fig. 5d). The performance parameters (short-circuit current density *J*_sc_, open-circuit voltage *V*_oc_, fill factor FF, and *η*) were extracted from the experimentally obtained *J-V* curves. The values of *η* and FF were determined using Eqs S2 and S3, respectively (see Supplementary Information).

The *J*_sc_ values obtained for the bare (reference) solar cell and for NGQD-coated CIGS (NGQD-CIGS) solar cells #3, #9, #11, and #12 were 29.71 and 31.61, 31.85, 29.59, and 32.09 mA/cm^2^, respectively. The relative change in the performance parameters is presented in Fig. 5(e). The relative changes in *J*_sc_ for NGQD-CIGS solar cells #3, #9, #11, and #12 was 6.4%, 7.19%, −0.41%, and 8.04%, respectively. The observed enhancement in *J*_sc_ for NGQD-CIGS solar cells #3, #9, and #12 is attributed to photon downconversion and light trapping. However, the *J*_sc_ value was slightly reduced for NGQD-CIGS solar cell #11. Further, a slight change in *V*_oc_ was observed for NGQD-CIGS solar cells #3, #9, #11, and #12 (relative changes: 0.92%, 0.93%, −0.09%, and 0.91%, respectively). NGQD-CIGS solar cells #3, #9, and #12 showed a slight improvement in the FF value. The overall enhancements in *η* for NGQD-CIGS solar cells #3, #9, and #12 were 7.60%, 10.49%, and 12.22%, respectively; however, a slight reduction in *η* was observed for NGQD-CIGS solar cell #11. Thus, the *η*_*dc*_ of NGQDs is mainly determined by the decay lifetimes at various *λ*_*em*_ values.

**Figure 6:**
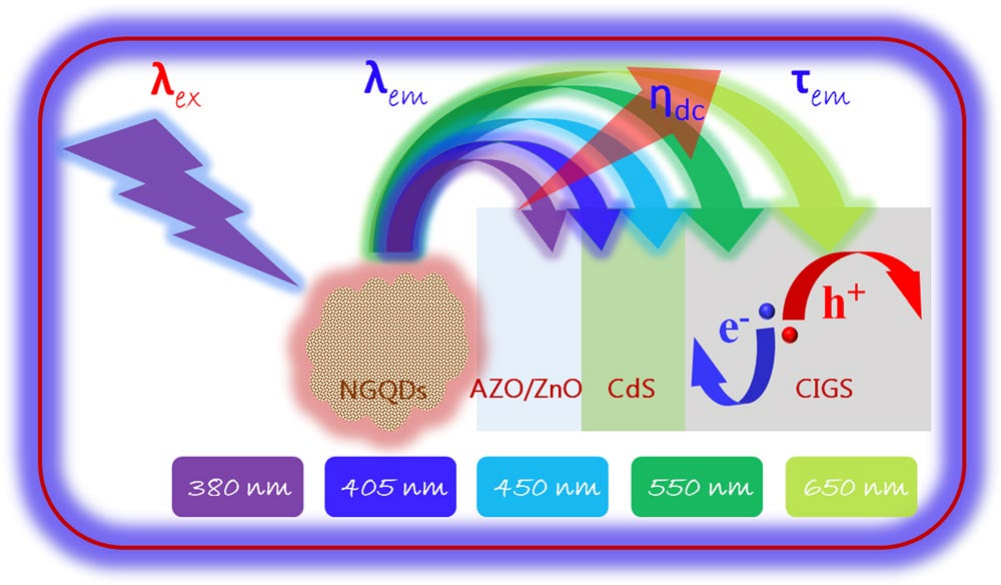
Schematic of the emission wavelength (λ_em_)-dependent PL decay lifetime (τ_em_) at λ_em_ = 379 nm, for the utilization of photons as charge carriers in NGQD-CIGS solar cells.

**Figure 7:**
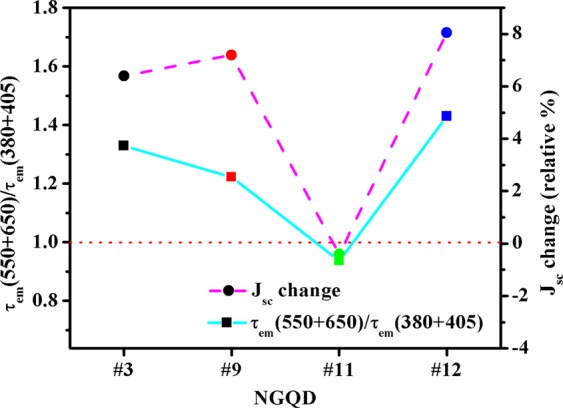
Ratio of the sum of τ_em_ values at higher λ_em_ (550 and 650 nm) to sum of τ_em_ values at lower λ_em_ (380 and 405 nm) NGQD samples and corresponding obtained J_sc_ values for NGQD-CIGS solar cells.

When any fluorescent material is excited with light with energy exceeding the corresponding band-gap, the light is absorbed and the electron moves to an excited state (a higher energy level). The excited electron attempts to return into its original position (initial energy level) by losing energy in the form of heat or light. Two main transitions involved in this process. First, the transition without light emission is called non-radiative recombination (fast band-to-band transition). Second, radiative recombinations occur during which light is emitted (due to slow extrinsic-effect-induced transitions)^40,41^. The usefulness of the emitted light can be determined by the average value, *τ*_*em*_. The energy-dependence lifetime of the emitted photons and their utilization as charge carriers in the NGQD-CIGS solar cells at *λ*_*ex*_ = 379 nm are shown in Fig. 6. The *η*_*dc*_ value is replicated in the obtained J_sc_ change (*ΔJ*_*sc*_). It can be seen that emitted light of various wavelengths can be absorbed in the different part of the CIGS solar cells. Light for which *λ*_*em*_ > 500 nm can easily be absorbed in the CIGS absorber, with most of these photons used for the current generation. However, a large fraction of the light for which *λ*_*em*_ < 500 nm absorbed in the ZnO/CdS layers is not used for the current generation. Therefore, the fraction of emitted light should be higher for cases in which *λ*_*em*_ > 500 nm to enhance the performance of the CIGS cells. Accordingly, *τ*_*em*_ should be higher for *λ*_*em*_ > 500 nm. Hence, for better utilization of the emitted photons in NGQD-CIGS solar cells, the values of *τ*_*em*_ for *λ*_*em*_ < 500 nm should be lower and the values of *τ*_*em*_ for *λ*_*em*_ > 500 nm should be higher. Photons for which *λ*_*em*_ ≈ 450 nm partially contribute to the generation of current. Therefore, the ratio (*R*_*em*_) of the sum of the τ_em_ values at higher values of *λ*_*em*_ (550 and 650 nm, *τ*_*em*_ > 500 nm) to the sum of *τ*_*em*_ values at lower values of *λ*_*em*_ (380 and 405 nm, *τ*_*em*_ < 500 nm) NGQD samples along with the corresponding obtained *ΔJ*_*sc*_ values for NGQD-CIGS solar cells are shown in Fig. 7. A trend similar to that of the variations of *R*_*em*_ and *ΔJ*_*sc*_ was obtained. No change in *J*_*sc*_ (*ΔJ*_*sc = *_0) was observed when *R*_*em*_* = *1 (as shown by the red dotted horizontal line). For cases in which *R*_*em*_ > 1, an enhancement in *J*_*sc*_ was obtained. However, *J*_*sc*_ was reduced slightly when *R*_*em*_ < 1 (NGQD-CIGS #11).

NGQDs with ultrahigh PL QYs (along with large *Δλ* values) was achieved by tuning the N and O moieties through control of the N-dopant concentration, synthesis temperature, and synthesis duration.

In this work, the important parameters influencing the down-conversion efficiency of the NGQDs are highlighted. In addition to the ultrahigh QY and large Stokes shift, the emission-wavelength-dependent PL decay lifetime plays a significant role in enhancing the down-conversion efficiency. The measured PL QYs of NGQD samples #3, #9, #11, and #12 were 78%, 99%, 80%, and 77%, respectively, and the corresponding calculated Stokes shifts were ~155, ~95, ~95, and ~95 nm. The observed enhancements in *η* for solar cells with NGQD samples #3, #9, and #12 were 7.60%, 10.49%, and 12.22%, respectively; however, NGQD-CIGS solar cell #11 exhibited a slightly lower *η* enhancement. NGQD-CIGS solar cell #12 exhibited the smallest *τ*_em_ at *λ*_*em*_ = 380 nm. By increasing the PL decay lifetime at *λ*_*em*_ > 500 nm, the CIGS solar-cell performance can be enhanced. This synthesis technology is economical and environmentally friendly. These low-cost processed NGQDs can be used to enhance the performance of the CIGS solar cells. The results of this study reveal that downconverters should have extremely low PL decay lifetime values at *λ*_*em*_ < 500 nm and high values at *λ*_*em*_ > 500 nm before they can be appropriately applied to CIGS solar cells.

## Materials and Methods

### Synthesis of NGQDs

First, an aqueous solution of GO (4.0 mg mL^−1^) was prepared by mixing GO powder in deionized (DI) water. An aqueous solution of PEI (0.5 g mL^−1^) was used as the N source. For the synthesis of NGQDs, PEI/GO solution mixtures were prepared with PEI/GO weight ratios of 10%, 20%, and 50% (denoted as PEI10, PEI20, and PEI50, respectively). Subsequently, GO was functionalized by stirring the PEI/GO solution mixture at 90 °C. Next, we synthesized NGQDs via heat treatment of the PEI/GO composites at various temperatures in an N_2_ environment for different durations. The products were then dispersed in ethanol and the solutions were filtered. The methodology used for the PL QY calculation is described in the Supplementary Information.

### Characterization of NGQDs

The absorbance spectra of the NGQDs were recorded using a UV–vis–near-infrared spectrophotometer (Carry-5000, Agilent Technologies). The PL spectra were recorded at excitation wavelengths (*λ*_ex_) of 379 and 405 nm using a PL spectrophotometer (Darsa, PSI Trading Co. Ltd.) with a Xe lamp. Time-resolved PL decay was measured via the time-correlated single-photon counting (TCSPC) technique using a time-resolved spectroscopy system (TRSS, Fluo Time 200, PicoQuant). A laser with an excitation wavelength of 379 nm was used to excite all of the NGQD samples. A photomultiplier tube (PMT) was used for the detection of the emitted light by the NGQD samples. To study the atomic structure of the NGQDs, HRTEM (HF-3300/NB5000/S-4800, Hitachi) was utilized. The elemental composition of the NGQDs was determined via XPS (ESCALAB 250Xi, Thermo Fisher Scientific). The XPS peaks were calibrated using the C1s peak. The Raman spectra were recorded using a confocal Raman spectrometer (Nicolet Almega XR, Thermo Fisher Scientific) with a *λ*_ex_ value of 532 nm.

### Solar-cell fabrication and characterization

The details of the CIGS solar-cell fabrication process are presented in the Supplementary Information. CIGS solar cells were initially fabricated, after which the synthesized NGQDs were coated onto the top of the solar cells. The *J*–*V* characteristics (active area of ~0.43 cm^2^) were measured under 1-sun AM1.5 G illumination using a source meter (Keithley 2400). The illumination intensity was calibrated using a Si reference solar cell (PV Measurements, USA). All measurements were performed at 25 °C.

## Supplementary information

Emission-wavelength-dependent photoluminescence decay lifetime of N-functionalized graphene quantum dot downconverters: Impact on conversion efficiency of Cu(In, Ga)Se2 solar cells
