# Supplementary material for: Emission-wavelength-dependent photoluminescence decay lifetime of N-functionalized graphene quantum dot downconverters: Impact on conversion efficiency of Cu(In, Ga)Se2 solar cells
Source: Sci Rep. 2019 Jul 25;9:10803. doi: 10.1038/s41598-019-47068-w (PMC6658560; doi:10.1038/s41598-019-47068-w)
Supplement: Supplementary file 1 — Emission-wavelength-dependent photoluminescence decay lifetime of N-functionalized graphene quantum dot downconverters: Impact on conversion efficiency of Cu(In, Ga)Se2 solar cells [file 41598_2019_47068_MOESM1_ESM.docx]

Supplementary Information

Emission-wavelength-dependent photoluminescence decay lifetime of N-functionalized graphene quantum dot downconverters: Impact on conversion efficiency of Cu(In, Ga)Se_2_ solar cells

Firoz Khan^1^ and Jae Hyun Kim^2^*

^1^Center of Research Excellence in Renewable Energy (CoRERE), King Fahd University of Petroleum and Minerals (KFUPM), Dhahran 31261, Saudi Arabia

^2^Smart Textile Convergence Research Group, Daegu Gyeongbuk Institute of Science & Technology (DGIST), 333 Techno Jungang-Daero, Hyeonpung-Myeon, Dalseong-Gun, Daegu 42988, Republic of Korea

^*^Corresponding author. Tel.: +82 53 7853610; fax: +82 53 785439

*E-mail address:* jaehyun@dgist.ac.kr (J.H. Kim)

**PL QY measurement**

The following equation was used to calculate the PL QY of the synthesized NGQDs^1^:

${QY}_{NQGD}= {QY}_{S}\left[ \left( \frac{\mu_{NGQD}}{\mu_{S}} \right)^{2}\left( \frac{A_{S}}{A_{NQGD}} \right)\left( \frac{I_{NGQD}}{I_{S}} \right) \right]$ , (1)

where *QY*_NGQD_ is the PL QY of the NGQDs, and *QY*_S_ = 58% (PL QY of a standard coumarin sample).

*A* = absorbance of the solution at *λ*_ex_

*μ* = refractive index of the solvent

*I* = integrated PL intensity

The subscripts “s” and “NGQD” refer to the standard (known QY) and NGQD, respectively.

**CIGS solar-cell fabrication**

Molybdenum-coated soda lime glass (SLG) was used as the substrate and back contact. The substrates were washed with acetone using sonication. In a typical process, a ~3-μm-thick CIGS layer was first deposited on the cleaned SLG substrate via a three-stage co-evaporation method^2^. Subsequently, a ~50-nm-thick CdS buffer layer was deposited on the CIGS layer via chemical bath deposition^3^. Next, intrinsic ZnO (i-ZnO) and Al-doped ZnO (ZnO:Al) films with thicknesses of ~50 and ~350 nm, respectively, were deposited on the CdS layer via direct-current magnetron sputtering. Finally, Ni (~50 nm) and Al (~1 μm) gridded front contacts were formed using electron-beam evaporation. NGQD-CIGS solar cells were fabricated by applying the NGQD samples on the front surface of the solar cells.

**Deposition of NQGDs**

First, the complete CIGS solar cells were washed with ethanol followed by drying in N_2_. The NGQDs solutions were prepared via the dispersion of NGQDs in ethanol. The NGQDs were coated on top of the complete CIGS solar cells using drop casting of NGQDs solution (20 µL/cm^2^). Finally, naturally dried in N_2_ ambient at room temperature.

**Calculation of average value of PL decay lifetime (τ_em_)**

In this case, the PL decay profile was acquired using time-resolved emission technique (time-correlated single-photon counting, TCSPC). Thus, the number of photons emitting with lifetime t is given by I_PL_.t. Therefore, the average value of τ_em_ was calculated using the following equation (S1)

$\tau_{em}=\frac{\int_{t1}^{t2} {(I}_{PL}.t)dt}{\int_{t1}^{t2} {(I}_{PL})dt}$ (S1)

Where t_1_ and t_2_ are the lower and upper limits on the time (t) axis. I_PL_ is the intensity of the PL decay curve.

**Calculation of conversion efficiency of CIGS solar cells (η)**

The *η* of the CIGS solar cell was calculated using the following equation (S2)

$\eta=\frac{J_{sc}V_{oc}FF}{P_{in}}$ (S2)

And the FF can be defined as

$FF=\frac{J_{m}V_{m}}{J_{sc}V_{oc}}$ (S3)

where P_in_ is incident illumination intensity, J_m_ and V_m_ are the current density and voltage at the maximum power point.


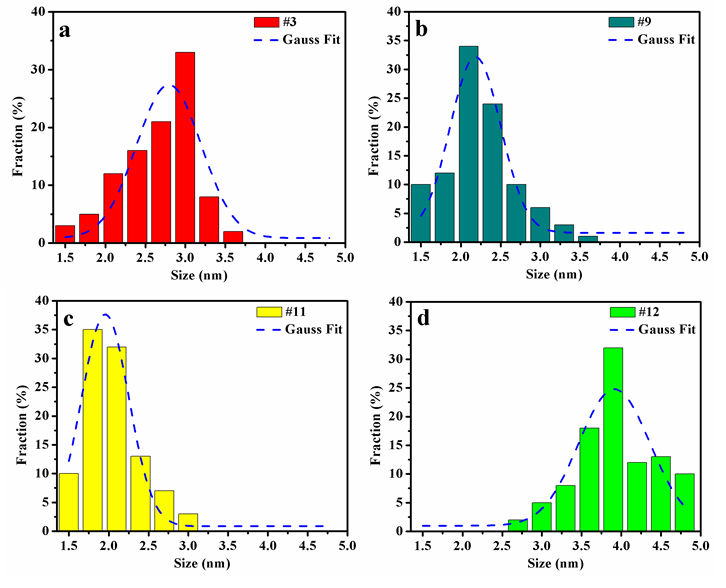


**Figure S1.** Size distribution and Gaussian fitting of NGQD samples (a) #3, (b) #9, (c) #11, and (d) #12.


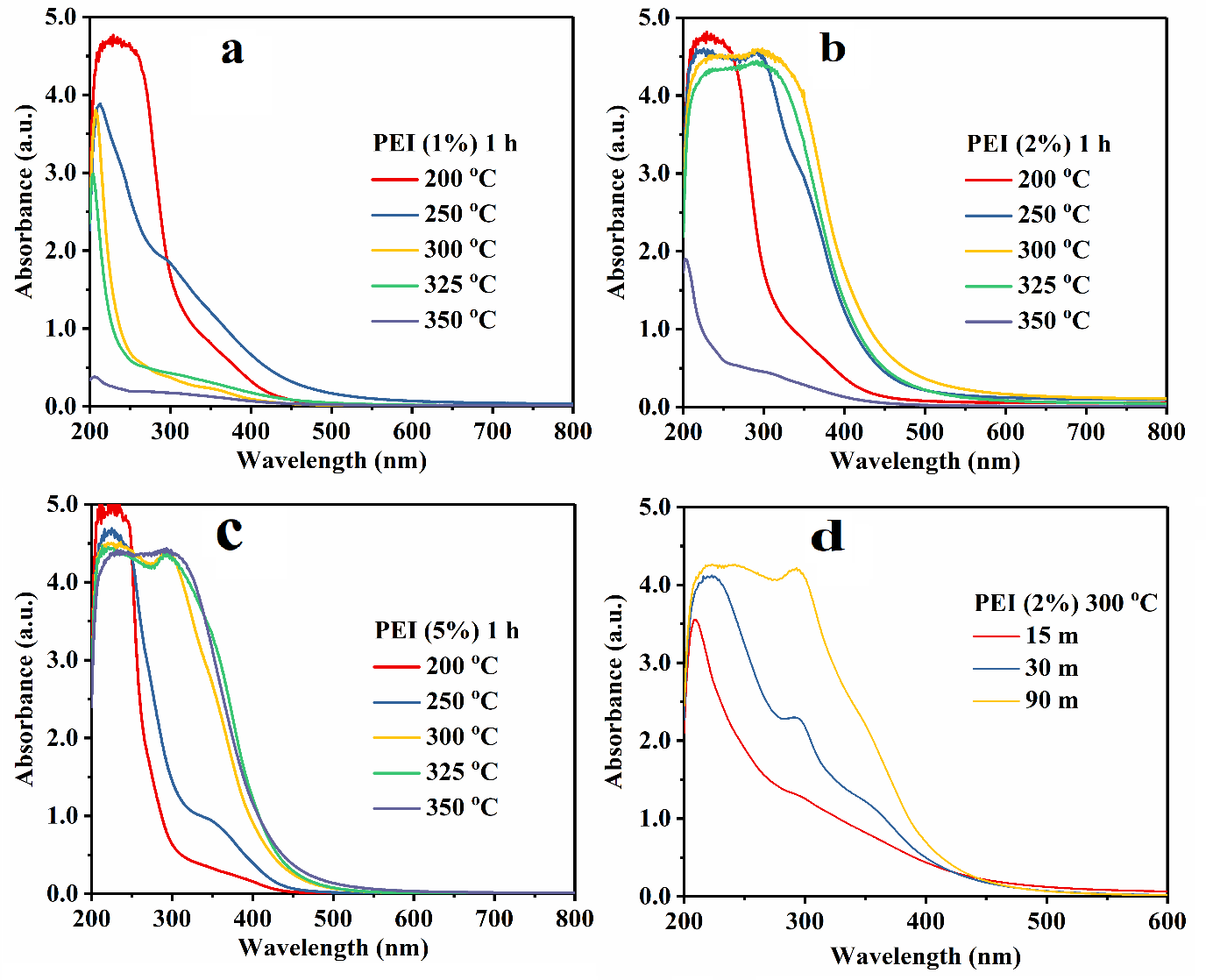


**Figure S2.** UV absorbance spectra of NGQD samples synthesized with PEI/GO weight ratios of (a) 10%, (b) 20%, and (c) 50% at temperatures ranging from 200 to 350 °C for 1 h. (d) UV absorbance spectra of NGQDs synthesized with the PEI/GO weight ratio of 20% at 300 °C for 15, 30, and 90 min.


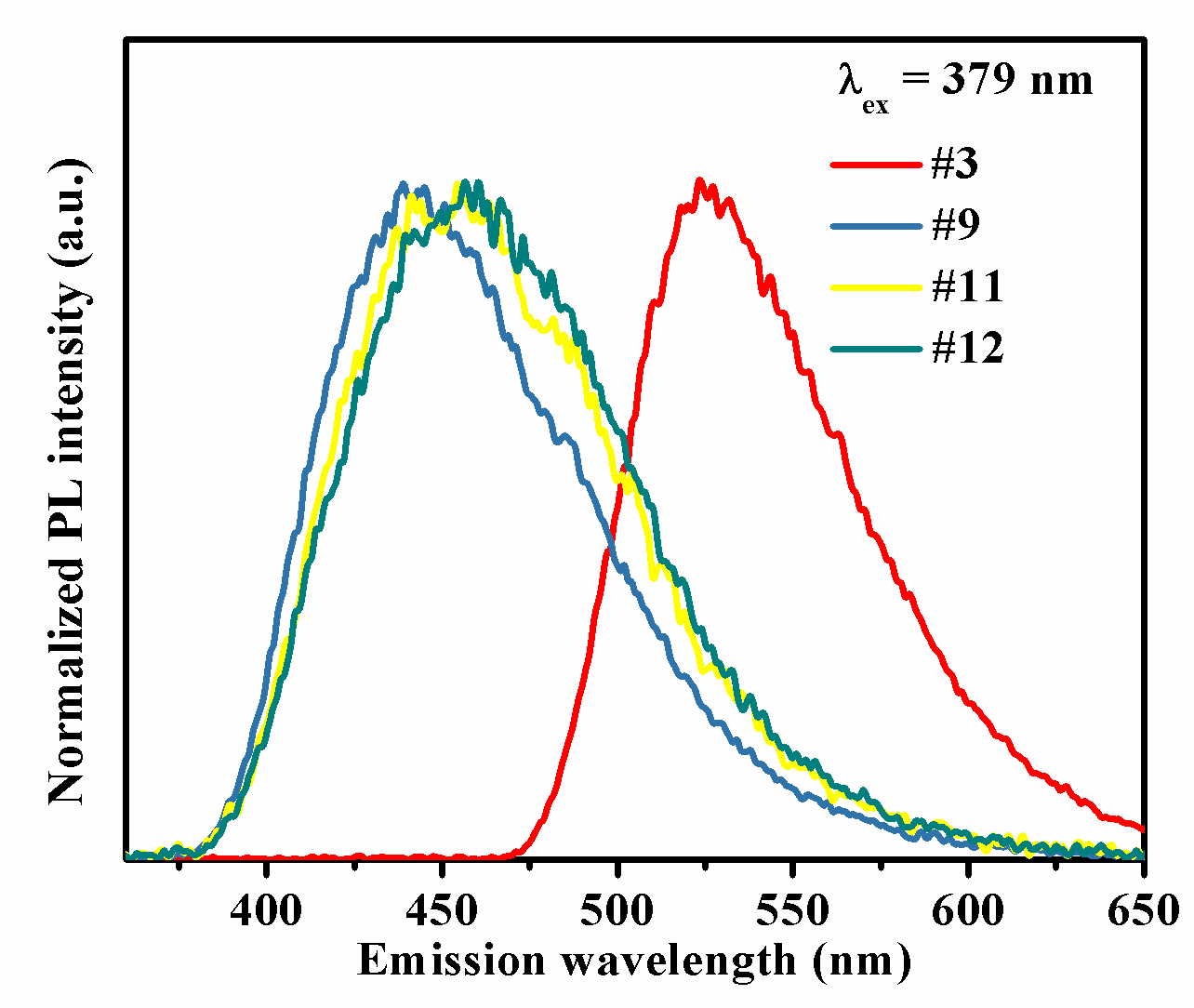


**Figure S3.** PL spectra emission spectra of NGQD samples #3, #9, #11, and #12 at λ_ex_ = 379 nm.


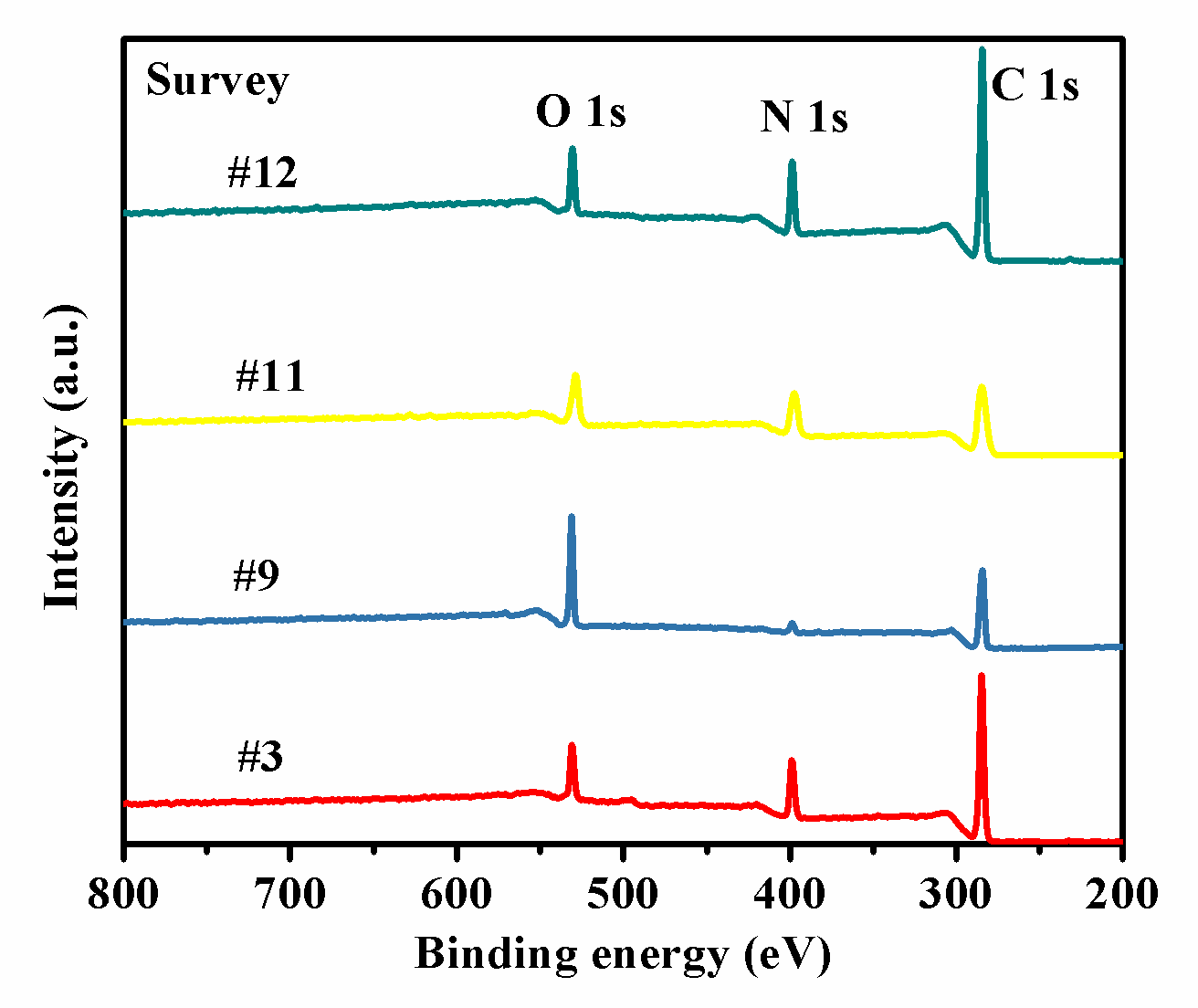


**Figure S4.** XPS survey spectra of NGQD samples #3, #9, #11, and #12.


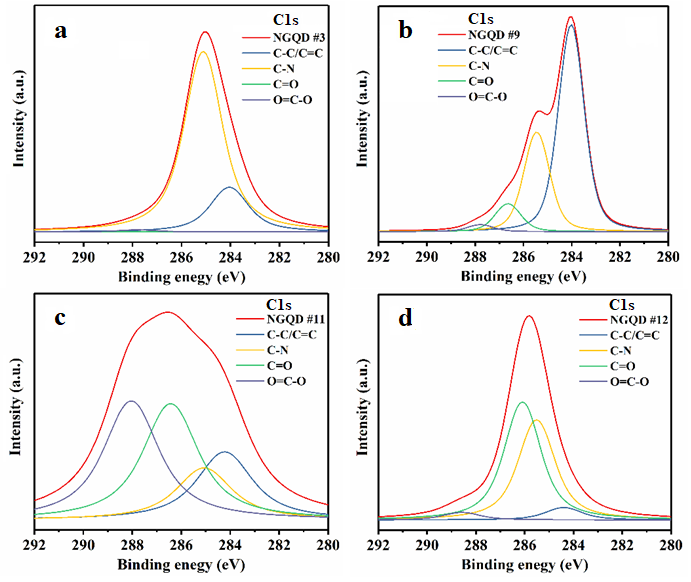


**Figure S5.** Deconvoluted C1s XPS spectra of NGQD samples (a) #3, (b) #9, (c) #11, and (d) #12.


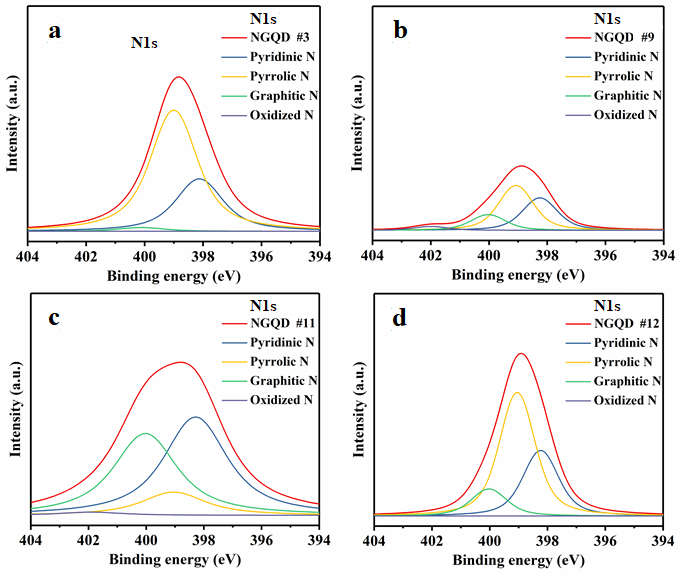


**Figure S6.** Deconvoluted N1s XPS spectra of NGQD samples (a) #3, (b) #9, (c) #11, and (d) #12.


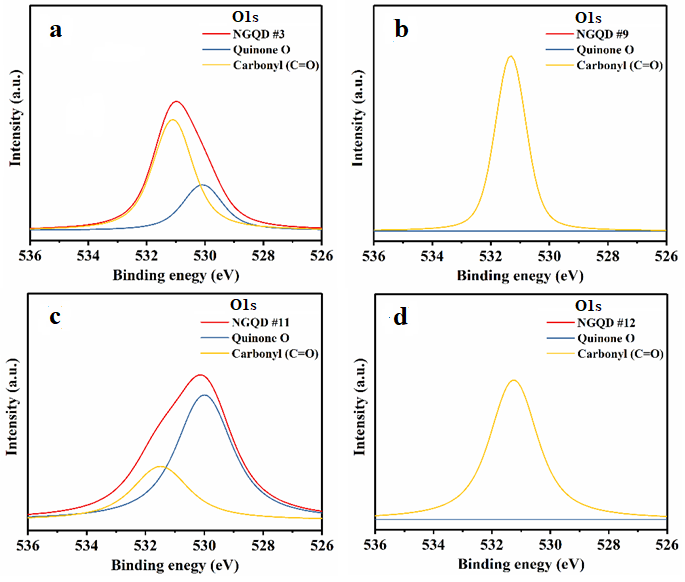


**Figure S7.** Deconvoluted O1s XPS spectra of NGQD samples (a) #3, (b) #9, (c) #11, and (d) #12.

**Table S1.** Synthesis parameters of various NGQD samples and the corresponding PL QY values obtained in this study.

| NGQDs | PEI/GO weight ratio | Temperature (°C) | Time (min) | PL QY (%) |
| --- | --- | --- | --- | --- |
| #1 | 20 | 325 | 60 | 21 |
| #2 | 20 | 300 | 15 | 27 |
| #3 | 50 | 350 | 60 | 78 |
| #4 | 10 | 325 | 60 | 45 |
| #5 | 50 | 300 | 60 | 51 |
| #6 | 10 | 350 | 60 | 48 |
| #7 | 10 | 280 | 60 | 48 |
| #8 | 20 | 300 | 30 | 55 |
| #9 | 20 | 300 | 60 | 99 |
| #10 | 20 | 350 | 15 | 73 |
| #11 | 10 | 250 | 15 | 80 |
| #12 | 20 | 250 | 60 | 77 |

**Table S2.** Comparison of the optical and photoluminescence properties of NGQDs synthesized in this study with those of previously reported NGQDs.

| Sample | Method | Absorption range (nm) | λ_ex_ (nm) | PL emission range/peak position | PL QY (%) | Reference |
| --- | --- | --- | --- | --- | --- | --- |
| NGQD | Solvothermal/hydrothermal process  Sonication 1 h  Autoclave heating at 200 °C for 5 h | 200–600 | 400 | 400–440/420 | 74 | 4 |
| NGQD | Solvothermal method using citric acid and ethanol diamine  160 °C for 4 h | 200–420 | 340–400 | 400–550/450 | 94 | 5 |
| NGQD | Solvothermal method using citric acid and diethylenetriamine  180 °C for 6 h | 200–420  200–420  200–420 | 350–430  460–540  480–560 | 400–550/450  450–650/550  500–750/580 | 90  29  22 | 6 |
| NGQD #3  NGQD #9  NGQD #11  NGQD #12 | Thermal method using GO and PEI  350 °C for 60 m  300 °C for 60 m  250 °C for 15 m  250 °C for 60 m | 200–450  200–450  200–450  200–450 | 405  405  405  405 | 550–650/560  460–650/500  460–650/500  460–650/500 | 78  99  80  77 | This work  7  This work  This work |

**Table S3.** Integrated area and corresponding τ_em_ at emission wavelengths of 380, 405, 450, 550 and 650 nm for NGQDs #3, #9, #11 and #12.

| Sample | λ_em_ (nm) | $\int_{t1}^{t2} {(I}_{PL}.t)dt$ | $\int_{t1}^{t2} {(I}_{PL})dt$ | $\tau_{em}=\frac{\int_{t1}^{t2} {(I}_{PL}.t)dt}{\int_{t1}^{t2} {(I}_{PL})dt}$ (ns) | Normalized τ_em_ (τ_em_/τ_em_ _@ λem = 550 nm_) |
| --- | --- | --- | --- | --- | --- |
| #3 | 380  405  450  550  650 | 8432  90503  116568  185985  137581 | 1945  15795  19353  27094  21120 | 4.33522  5.72985  6.02325  6.86443  6.51425 | 0.63155  0.83472  0.87746  1  0.94899 |
| #9 | 380  405  450  550  650 | 22184  85137  113827  173927  133416 | 4264  14839  18791  25397  20459 | 5.20263  5.73738  6.05753  6.84833  6.52114 | 0.75969  0.83778  0.88453  1  0.95222 |
| #11 | 380  405  450  550  650 | 51000  254017  253856  255825  164967 | 6762  29308  30951  32440  22585 | 7.54215  8.66716  8.20187  7.8861  7.30427 | 0.95638  1.09904  1.04004  1  0.92622 |
| #12 | 380  405  450  550  650 | 2521  152490  174974  227679  151170 | 888  21576  24577  31009  22154 | 2.83896  7.06758  7.11942  7.34235  6.8236 | 0.38666  0.96258  0.96964  1  0.92935 |

**References**

1. http://www.fluortools.com/software/ae/documentation/qy.
2. Couzinie-Devy, F., Barreau, N. & Kessler, J. Re-investigation of preferential orientation of Cu(In, Ga)Se_2_ thin films grown by the three-stage process. *Prog. Photovolt.* **19**, 527–536 (2011).
3. Khan, F., Lee, H. J., Oh, M. & Kim, J. H. Analysis of photovoltaic cell parameters on non-vacuum solution process Cu(In, Ga)Se_2_ thin film based solar cells. *Sol. Energy* **108**, 189–198 (2014).
4. Sun, J. *et al*. Ultra-high quantum yield of graphene quantum dots: aromatic-nitrogen doping and photoluminescence mechanism. *Part. Part. Syst. Charact.* **32**, 434–440 (2015).
5. Que, D. *et al*. Formation mechanism and optimization of highly luminescent N-doped graphene quantum dots. *Sci. Rep.* **4**, 5294-1–5294-9 (2014).
6. Qu, D., Zheng, M., Xie, Z., & Sun, Z. Tailoring color emission from N-doped graphene quantum dots for bioimaging application. *Light: Sci. Appl.* **4**, e364-1–e364-8 (2015).
7. Khan, F., Kim, J. H. N-functionalized graphene quantum dots with ultrahigh quantum yield and large Stokes shift: efficient downconverters for CIGS solar cells. *ACS Photon*. 5, 4637-4643 (2018).
